# Splice Variants of Superoxide Dismutases in Rice and Their Expression Profiles under Abiotic Stresses

**DOI:** 10.3390/ijms22083997

**Published:** 2021-04-13

**Authors:** Ajay Saini, Jai S. Rohila, Ganesan Govindan, Yong-Fang Li, Ramanjulu Sunkar

**Affiliations:** 1 Department of Biochemistry and Molecular Biology, Oklahoma State University, Stillwater, OK 74078, USA; ajays@barc.gov.in (A.S.); ganesan.govindan@okstate.edu (G.G.); yongfang.li@okstate.edu (Y.-F.L.); 2 Bhabha Atomic Research Centre, Molecular Biology Division, Trombay, Mumbai, Maharashtra 400085, India; 3 Homi Bhabha National Institute, Anushaktinagar, Trombay, Mumbai, Maharashtra 400094, India; 4 Dale Bumpers National Rice Research Center, United States Department of Agriculture-Agricultural Research Services, Stuttgart, AR 72160, USA; jai.rohila@usda.gov

**Keywords:** abiotic stress, alternative splicing, rice, splice variants, SODs, superoxide dismutases

## Abstract

The superoxide dismutases (SODs) play vital roles in controlling cellular reactive oxygen species (ROS) that are generated both under optimal as well as stress conditions in plants. The rice genome harbors seven SOD genes (*CSD1*, *CSD2*, *CSD3*, *CSD4*, *FSD1*, *FSD2,* and *MSD*) that encode seven constitutive transcripts. Of these, five (*CSD2*, *CSD3*, *CSD4*, *FSD1,* and *MSD*) utilizes an alternative splicing (AS) strategy and generate seven additional splice variants (*SVs*) or mRNA variants, i.e., three for *CSD3*, and one each for *CSD2*, *CSD4*, *FSD1,* and *MSD*. The exon-intron organization of these *SVs* revealed variations in the number and length of exons and/or untranslated regions (UTRs). We determined the expression patterns of *SVs* along with their constitutive forms of *SODs* in rice seedlings exposed to salt, osmotic, cold, heavy metal (Cu^+2^) stresses, as well as copper-deprivation. The results revealed that all seven *SVs* were transcriptionally active in both roots and shoots. When compared to their corresponding constitutive transcripts, the profiles of five *SVs* were almost similar, while two specific *SVs* (*CSD3-SV4* and *MSD-SV2*) differed significantly, and the differences were also apparent between shoots and roots suggesting that the specific SVs are likely to play important roles in a tissue-specific and stress-specific manner. Overall, the present study has provided a comprehensive analysis of the SVs of SODs and their responses to stress conditions in shoots and roots of rice seedlings.

## 1. Introduction

In eukaryotes, splicing of intron-containing precursor messenger RNAs (pre-mRNAs) generates mature mRNA [1,2,3]. Interestingly, splicing also provides an opportunity for generating diverse mature transcripts with alternative combinations of exons/untranslated regions (UTRs) via specific events, such as the intron retention, exon skipping, alternative splice sites, and mutually exclusive exons, by a process known as ‘alternative splicing’ (AS) [4]. AS enhances the transcriptome and proteome diversity with altered stability, properties, and subcellular localization in an organism that can affect a variety of cellular processes [3,5,6]. Previous studies have estimated that more than 60% of the intron containing pre-mRNAs in Arabidopsis undergo AS [7,8]. The recent expansion of RNA-Seq datasets has emphasized the importance of AS in plants [3,7,9]. Although high-throughput RNA-seq analysis reveals a global view of AS events, it is important to investigate their spatial and temporal expression profiles in more detail.

Reactive oxygen species (ROS), such as singlet oxygen (^1^O_2_), superoxide radical (O_2_^•–^), hydrogen peroxide (H_2_O_2_), and hydroxyl radical (^•^OH), are continually generated in cells due to various metabolic processes in plants. Their tight regulation is physiologically important due to the beneficial roles they play in a variety of essential redox-related cellular processes [10,11]. On the other hand, ROS tend to accumulate in excess amounts, specifically in the chloroplast and mitochondria, under a variety of abiotic stress conditions, such as drought, salinity, low and high temperatures, high light, metal pollutants, ozone, UV radiation, and nutrient starvation [12,13,14,15,16]. Excessive ROS levels can cause irreversible damage to lipids, proteins, DNA and alter the redox state, all of which can cause cell death. To maintain ROS homeostasis both under control as well as stress conditions, plants utilize enzymatic (superoxide dismutases, catalases, and peroxidases) and non-enzymatic (glutathione and other thiol-containing compounds, ascorbic acid, anthocyanin, etc.) defense systems that can decrease ROS levels either by scavenging or undergoing oxidation themselves to decrease the oxidation of macromolecules [13,17]. Of the enzymatic systems, superoxide dismutases (SODs) establish the first line of defense against ROS accumulation by catalyzing the conversion of O_2_^•–^ into O_2_ and H_2_O_2_. Based on the metal cofactor present at the active site, SODs are categorized into CuZn-SOD, Fe-SOD, Mn-SOD, and Ni-SOD [18]. Unlike in animals, SODs are found to be encoded by multiple genes in plant genomes [19,20]. Multiple layers of gene regulatory processes ranging from transcriptional, post-transcriptional to post-translational levels contribute to the SOD abundances, which may be affected differently under different stress conditions. Here, we focused on analyzing splice variants (SVs) of rice SODs, which is one aspect of post-transcriptional regulation under diverse abiotic stresses.

Thus far, only a few SVs of SODs are known in plants. An enzymatically active SV of Fe-SOD from rice that retained intron-4 [21]; an *SV* of *SOD* from *Populus trichocarpa*, whose exon-6 has 69 extra bp [22]; an *SV* of *CSD2* (*NSP-GmCSD2*) from soybean that retained intron-4 (100 bp) but contained a premature stop codon [23]; and an *SV* of *FSD3* (*FSD3S*) from Arabidopsis that retained two introns (6 and 7), but included a stop codon at an earlier position compared with the constitutive *FSD3* [24]. Other than these, very little information is available on AS of SODs in plants. A comprehensive analysis of SVs of SODs is essential for a better understanding of ROS homeostasis under normal and stress conditions. In rice, there are seven SODs, including four CuZn-SODs (cytosolic CSD1: Os03g22810; chloroplastic CSD2: Os08g44770; putative peroxisomal CSD3: Os03g11960; and cytosolic CSD4: Os07g46990), two chloroplastic Fe-SODs (FSD1: Os06g05110, and FSD2: Os05g02500), and a mitochondrial Mn-SOD (MSD: Os05g25850). The available exon-intron organization of rice SODs and their SVs at the Rice Genome Annotation Project (RGAP) suggested that five predicted SOD loci (CSD2, CSD3, CSD4, FSD1, and MSD) may generate a total of seven SVs (http://rice.plantbiology.msu.edu/). In the present study, we attempted to characterize *SVs* of all *SODs* in rice. By using RT-qPCR assays, the expression profiles of the seven constitutive *SOD* transcripts as well as their seven *SVs* were determined in roots and shoots of seedlings exposed to different abiotic stresses such as the salt (150 mM NaCl), osmotic (15% PEG-6000), cold (4 °C), and heavy metal (100 μM Cu^+2^) stress including copper-deprivation. The results revealed that majority of the *SOD* SVs are relatively abundant and exhibited largely similar profiles as their constitutive transcripts but a few displayed opposite profiles in a tissue-specific and stress-specific manner.

## 2. Results

### 2.1. Analysis of UTR, Intron, and Exon Organization of the SOD SVs

**Figure 1:**
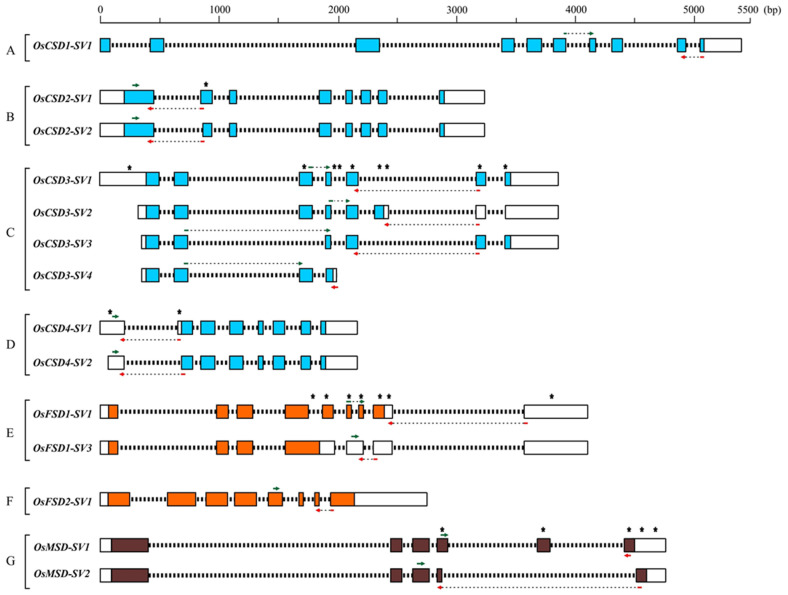
Schematic representation of splice variant transcripts of rice CuZn, Fe, and Mn-SODs as available at the Rice Genome Annotation Project (RGAP) website (http://rice.plantbiology.msu.edu/). (A) LOC_Os03g22810 (*CSD1*), (B) LOC_Os08g44770 (*CSD2*), (C) LOC_Os03g11960 (*CSD3*), (D) LOC_Os07g46990 (*CSD4*), (E) LOC_Os06g05110 (*FSD1*), (F) LOC_Os06g02500 (*FSD2*) and (G) LOC_Os05g25850 (*MSD*). The three types of SODs are represented by different colors, blue (CuZn-SODs), orange (Fe-SODs), and brown (Mn-SOD). Scale on the top indicates the relative sizes of the genes and positions of exons and introns. Colored boxes represent exons, white boxes represent untranslated regions (UTRs), and thick black dashed lines represent intronic regions. Forward and reverse primer binding regions are indicated by green and red arrows (the thin dashed line connecting the arrowhead and tail indicates the continuity of the primers designed at the exon-exon/UTR junction). Asterisk (*) indicates the exon/UTR regions that show heterogeneity between the splice variants of a gene.

A survey of the Nipponbare rice genome identified a total of seven *SOD* genes, which were further categorized into *CSDs* (*CuZn-SODs*), *FSDs* (*Fe-SODs*), and *MSD* (*Mn-SOD*) based on the presence of a metal cofactor in the respective proteins (Table 1). Previous reports included Os04g48410 as a putative *CuZn-SOD* and claimed eight *SOD* genes in the rice genome [25,26]. However, Os04g48410 codes for copper chaperone that delivers copper to CuZn-SOD proteins [27]. Of the seven *SOD* genes, four code for *CSDs*, two for *FSDs*, and one for *MSD*. It is known that the majority of multiple exon-containing genes may undergo AS during pre-messenger RNA processing and generate a more complex transcriptome and, consequently, the proteome. The Rice Genome Annotation Project predicted seven *SVs* for five genes, i.e., three *SVs* for *CSD3* (Os03g11960), one *SV* each for *CSD2* (Os08g44770), *CSD4* (Os07g46990), *FSD1* (Os06g05110), and *MSD* (Os05g25850). A schematic presentation of *SVs* of the *SOD*s with respect to the presence or absence of exons/introns and their length heterogeneity at respective UTRs (5′ and 3′ ends) is shown in Figure 1.

Among the four *CSD* genes, *CSD1* does not appear to undergo AS (Figure 1A). The two *SVs* of *CSD2* have eight exons. However, the coding sequence of exon-2 in *SV2* was 12 bp smaller at 5′end when compared to *SV1* (Figure 1B). The *CSD3* showed maximum heterogeneity among all *SODs,* and there were four *SVs* (*SV1*, *SV2*, *SV3*, and *SV4*) due to varying combinations of UTRs, exons, and introns (Figure 1C). The *CSD3-SV2* has a shorter 5′UTR and contained an alternative exon at the sixth position (E-6) that harbored a coding region as well as a part of 3′ UTR. The E-7 and E-8 exons were part of the considerably long 3′ UTR in this *SV*. The *CSD3-SV3* was similar in organization to canonical *CSD3-SV1* but harbored a shorter 5′ UTR and skipped the E-3 (exon-3). The *CSD3-SV4* was the smallest *SV* of this gene; its 5′ UTR was similar to *SV3*, the first three exons (E-1, E-2, and E-3) were similar to those of the *SV1*, the constitutive transcript, and the E-4 had an extra-long coding region, and an extremely short 3′ UTR generated by partial retention of the intron between E-4 and E-5. (Figure 1C). Interestingly, *CSD3-SV3* appears to be missing exon encoding Zn-binding site. The *OsCSD4* encoded two *SVs* with a similar organization of exons but differed in length of the 5′ UTR, localized in exon-2 (Figure 1D).

Among the two *FSD* genes, the LOC_Os06g05110 coding for the constitutive transcript (*FSD1-SV1*) contained a 5′ UTR, eight exons, and a long 3′ UTR. The *OsFSD1* was alternatively spliced, resulting in generation of *SV3* (Figure 1E). The *FSD1-SV3* was generated by two intron retention events (between E-4 and E-5 and between E-6 and E-7) and contained a relatively short coding region compared to the constitutive form, the *FSD1*. The exons beyond E-4 constituted the substantially long 3′ UTR region. The second *FSD*, *FSD2,* was found to have only constitutive transcript without SVs (Figure 1F).

A mitochondrial *Mn-SOD*, *MSD,* has a constitutive transcript (*OsMSD-SV1*) and an SV (*OsMSD-SV2*). The alternatively spliced *MSD-SV2* possessed five exons, whereas the constitutive transcript (*MSD-SV1*) contains six exons. The *MSD-SV2* completely lacked E-5 and some regions from E-4 and E-6 and a portion of the 3′ UTR (Figure 1G). Taken together, the *SVs* of *SODs* in rice differed substantially both in their UTRs and coding regions.

### 2.2. The Basal Expression Levels of SVs of SOD Transcripts

**Figure 2:**
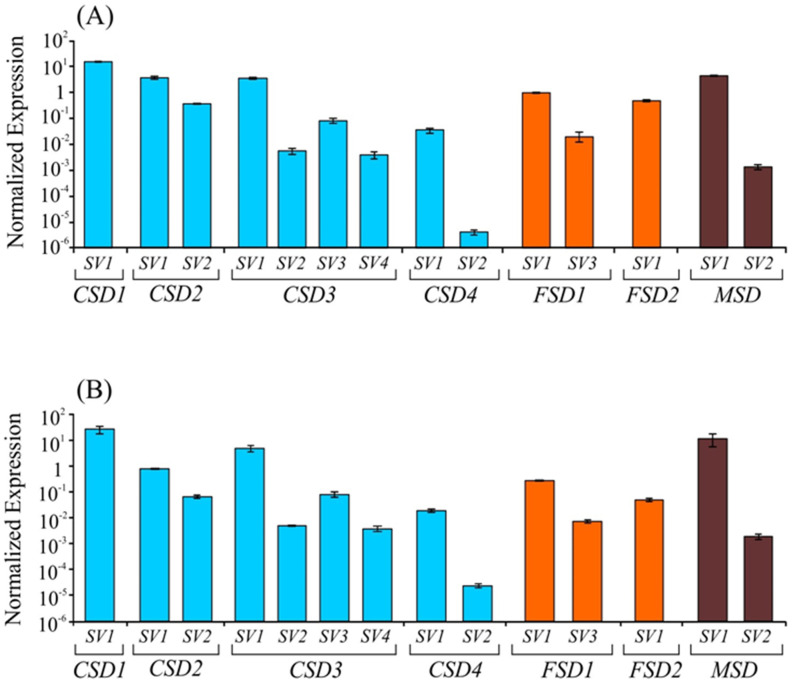
Basal relative transcript abundance of various splice variants of *CSDs*, *FSDs*, and *MSD* in shoot (**A**) and root (**B**) tissue. The transcript abundances shown on *Y*-axis were normalized using OsActin as a reference gene as per Schmittgen and Livak [28]. The data is presented as mean normalized transcript level ± SD of two repeats of independent biological replicates (each biological replicate had three technical replicates).

To assess the relative transcript abundance of the *SVs* of different *SODs* in shoot and root tissues, RT-qPCR analysis was carried out using *SV*-specific primers to distinguish each *SV* from its constitutive transcript (Figure 1 and Table S1). The expression of major transcripts of all seven *SOD* genes as well as seven *SVs* from the five *SOD* genes were detected in both shoot and root tissues (Figure 2). Among the *CSD*, *FSD*, and *MSD*s, transcripts from *CSD*s contributed the most to the total *SOD* transcript abundances.

Among the constitutive transcripts of *SODs*, *CSD1* showed the highest abundances in both shoots and roots (Figure 2; Supplementary materials Figure S1). As expected, the chloroplastic *CSD2* showed greater transcript abundances in shoots compared to roots (Figure 2B). The constitutive transcript levels of *CSD3* (*CSD3-SV1*) and *CSD2* (*CSD2-SV1*) were relatively greater compared to the *CSD4* (*CSD4-SV1*) levels both in roots and shoots. The constitutive transcript levels of both the *FSD*s (*FSD1-SV1* and *FSD2-SV1*) and the mitochondrial *MSD* (*MSD-SV1*) were almost similar between shoot and root tissues (Figure 2A,B). In general, the abundances of *SVs* in roots and shoots were relatively lower compared to their constitutive transcripts of *SOD*s (Figure 2). The expression profiles of *OsCSD4-SV2* did not show a satisfactory level of amplification potentially due to extremely low abundances, therefore, were not analyzed and included in further analyses.

### 2.3. Expression Profiles of SODs and Their SVs under Abiotic Stresses

Given their critical roles under stress conditions, it is important to determine the expression profiles of different *SVs* of *SODs* under diverse stress conditions. Here we report the expression profiles of *SVs* of the *SODs* in shoot and root tissues of rice seedlings exposed to five different abiotic stresses viz. salt (150 mM NaCl), osmotic (15% PEG-6000), cold (4 °C), and heavy metal (100 μM Cu^+2^), and copper-deprivation.

#### 2.3.1. Salt Stress

**Figure 3:**
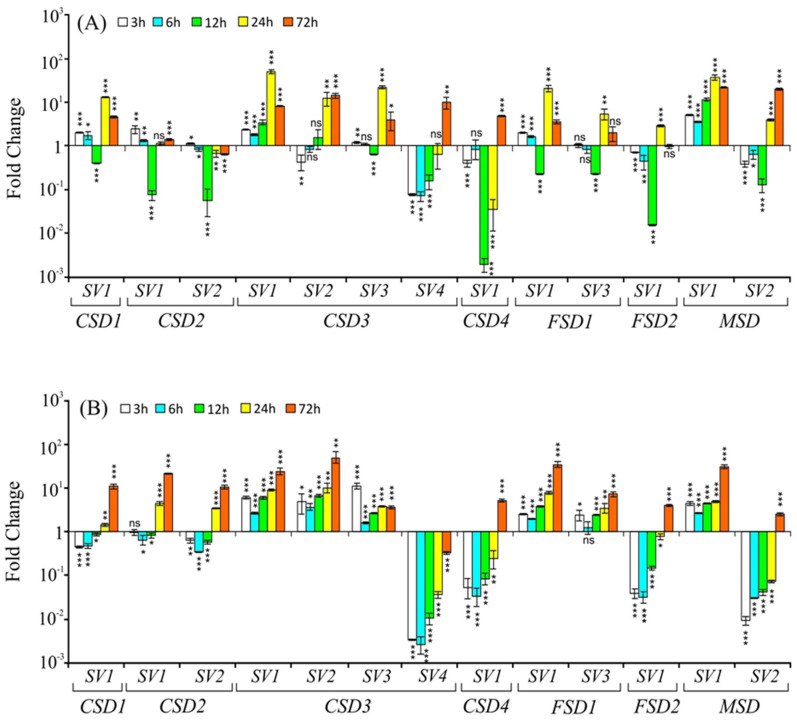
Relative transcript levels (on *Y*-axis) of *CSDs*, *FSDs*, and *MSD* splice variants (indicated by numbers on *X*-axis) in response to salt stress (150 mM NaCl), in shoot (**A**) and root (**B**) tissue at 3 h, 6 h, 12 h, 24 h, and 72 h after the stress treatment. Transcript levels in control and treated samples were normalized against OsActin, and fold change (relative expression) was estimated as per Schmittgen and Livak [28] using Actin as a reference gene. The experiment was carried out with two independent biological replicates (each sample had three technical replicates), repeated twice, and data are presented as mean transcript level ± SD. The significance level is indicated by * *p* < 0.05, ** *p* < 0.01, *** *p* < 0.001, and ns (no significant difference).

The transcript profiles of both the constitutive form of *SODs* and their *SVs* were found to be altered under salt stress (Figure 3). *CSD1-SV1* was upregulated during initial time points (3 h and 6 h) of salt exposure but temporarily downregulated at 12 h and upregulated again at later time points in both the shoots and the roots. The constitutive transcript *CSD2-SV1* and its *SV* (*CSD2-SV2*) profiles displayed variable responses; mild upregulation during initial time points (3 h and 6 h) and later time points (24 h and 72 h) but strongly downregulated at 12 h of stress in shoots. In roots, the levels were initially downregulated until 12 h and then upregulated at 24 h and 72 h. Both in shoots and roots, the constitutive transcript levels of *CSD3* (*CSD3-SV1*) were significantly upregulated at all time points. On the other hand, two *SVs* of *CSD3* (*SV2* and *SV3*) showed initial downregulation until 12 h, followed by significant upregulation at 24 h and 72 h in shoots. In roots, both the *SVs* showed a similar pattern (significantly upregulated at all time points) as that of the constitutive transcript (*CSD3-SV1*). Remarkably, unlike the constitutive *CSD3-SV1,* whose levels were significantly upregulated at all time points, the *CSD3-SV4* showed substantial downregulation in shoots at 3 h, 6 h, and 12 h time points but displayed significant upregulation at 72 h. In roots, *CSD3-SV4* levels significantly declined at all time points (Figure 3B). The *CSD4-SV1* levels were downregulated until 24 h and then upregulated at 72 h both in shoots and roots.

The constitutive *FSD1* transcript (*FSD1-SV1*) and its *SV* (*FSD1-SV3*) showed a duration-dependent increase in roots. However, in shoots, their levels showed different profiles; initially, small upregulation followed by a downregulation, and then upregulation at later time points, approximately similar to *CSD1-SV1* profiles (Figure 3A,B). By contrast, the *FSD2-SV1* levels showed variable responses at different time points, largely downregulated initially in both shoots and roots and then upregulated (Figure 3A,B). The constitutive form of *MSD* (*MSD-SV1*) showed significant upregulation in both shoots and roots at all time points tested while its *SV* (*MSD-SV2*) in shoots showed an initial downregulation largely until 12 h and then upregulation at 24 h and 72 h, whereas its levels in roots were significantly downregulated until 24 h and then upregulated at 72 h (Figure 3A,B). Notably, the *CSD1-SV1*, *FSD1-SV1,* and *FSD1-SV3* displayed similar profiles in shoots under salt stress.

#### 2.3.2. Osmotic Stress

**Figure 4:**
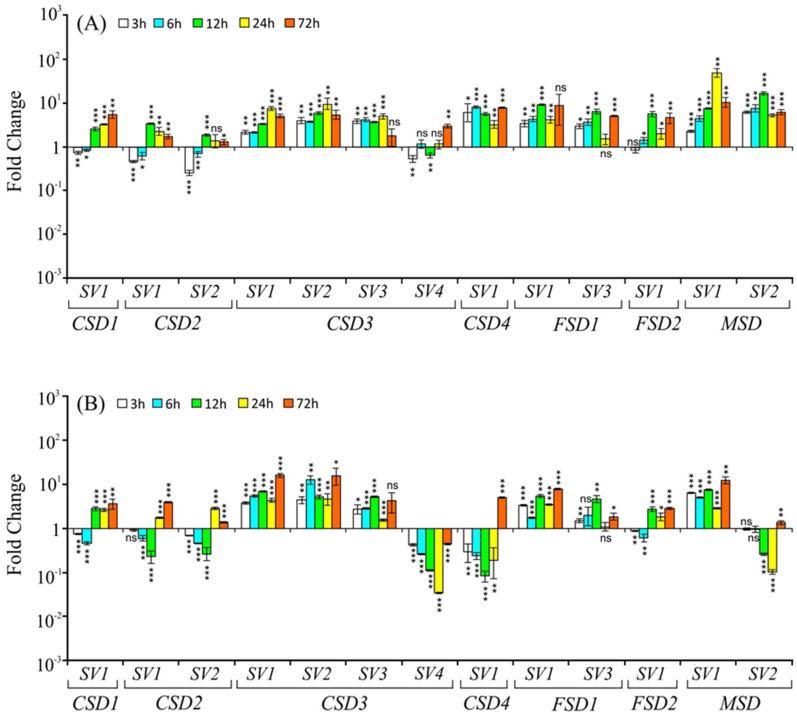
Relative transcript levels (on *Y*-axis) of *CSDs*, *FSDs*, and *MSD* splice variants (indicated by numbers on *X*-axis) in response to osmotic stress (15% PEG), in shoot (**A**) and root (**B**) tissue at 3 h, 6 h, 12 h, 24 h, and 72 h after the stress treatment. For normalization and statistical analysis, see figure legend 3.

In shoots, the transcript levels of *CSD1-SV1* and *CSD2-SV1* (constitutive transcripts) were initially downregulated (3 h and 6 h) and then significantly upregulated at later time points of PEG-induced osmotic stress (Figure 4A). The *SV* of *CSD2* (*CSD2-SV2*) profiles were very similar to *CSD2-SV1*, its constitutive transcript. The transcript levels of *CSD3 –SV1*, *-SV2,* and *-SV3* were upregulated while *-SV4* showed variable responses: downregulated at 3 h and 12 h but upregulated at 72 h (Figure 4A). The *CSD4-SV1*, *FSD1-SV1*, *FSD1-SV3*, *FSD2-SV1*, *MSD-SV1,* and *MSD-SV2* transcript levels were upregulated at all time points in shoots (Figure 4A). However, in roots, the expression profiles were slightly varied in several cases. For instance, *CSD1-SV1* showed mild downregulation during initial time points (until 6 h), followed by upregulation at later time points. Both the *CSD2-SV1* (constitutive form) and *CSD2-SV2* showed a similar pattern of regulation (downregulation until 12 h followed by upregulation) in response to the osmotic stress (Figure 4B). Of the four *SVs* of *CSD3*, three *SVs* (*SV1, SV2,* and *SV3*) were upregulated, while the *SV4* was downregulated, similar to its profiles in shoots. The *CSD4-SV1* also showed downregulation until 24 h and then upregulation at 72 h (Figure 4B). Both *SVs* of *FSD1* (*SV1* and *SV3*) exhibited upregulation in roots, although the regulation of *FSD1-SV3* was less apparent under osmotic stress. The *FSD2-SV1* was initially downregulated (3 h and 6 h) and then upregulated at later time points. Interestingly, *MSD SVs* displayed contrasting profiles for most of the time points, i.e., *MSD-SV1* levels were significantly upregulated at all time points, whereas the *MSD-SV2* levels were unaltered during the initial phase (up to 6 h) and then downregulated at 12 h and 24 h and then mildly but significantly upregulated at 72 h in roots under osmotic stress (Figure 4B).

#### 2.3.3. Cold Stress

**Figure 5:**
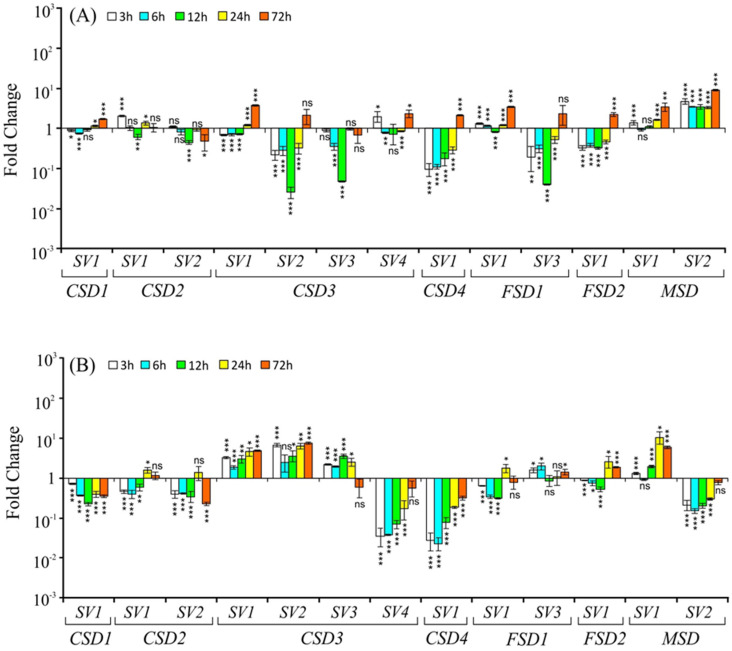
Relative transcript levels (on *Y*-axis) of *CSDs*, *FSDs*, and *MSD* splice variants (indicated by numbers on *X*-axis) in response to cold stress (4 °C), in shoot (**A**) and root (**B**) tissue at 3 h, 6 h, 12 h, 24 h, and 72 h after the stress treatment. For normalization and statistical analysis, see figure legend 3.

Under cold stress, *SVs* of *CSD1*, *CSD4*, *CSD3* (*SV1*, *SV2,* and *SV4*), *FSD1* (*SV1* and *SV3*), and *FSD2* transcript levels were largely either remained unaltered or downregulated until 24 h and then slightly upregulated at 72 h in shoots (Figure 5A). The degree of downregulation was strong in the case of *CSD3-SV2*, *CSD3-SV3*, *CSD4-SV1*, *FSD1-SV3*, and *FSD2-SV1*. The *MSD-SV1* levels were mildly but significantly upregulated at 3 h, 24 h, and 72 h, whereas its *SV* (*MSD-SV2*) transcripts were significantly upregulated at all time points under cold stress in shoots (Figure 5A).

In roots, the *CSD1-SV1*, *CSD4*, both *SVs* of *CSD2* as well as *CSD3-SV4* levels were downregulated throughout the duration of the stress treatment, while *CSD3-SV1*, *CSD3-SV2,* and *CSD3-SV3* levels were upregulated. The *FSD1* and *FSD2* levels altered, but only mildly. On the other hand, *MSD-SV1* levels in roots were significantly upregulated, especially at later time points (12 h, 24 h, and 72 h), whereas *MSD-SV2* levels were largely significantly downregulated under cold stress (Figure 5B).

#### 2.3.4. Heavy Metal (Copper) Stress and Copper-Deprivation

Heavy metals can be grouped into redox-active (ex., Cu, Fe, Cr, and Co) and redox non-active (ex., Zn and Cd) metals. The redox-active heavy metals contribute to the cellular redox reactions (reduced Cu^+^ and oxidized Cu^2+^ states), which could generate ^•^OH and H_2_O_2_ via Haber–Weiss and Fenton reactions that, in turn, can initiate lipid peroxidation [29]. In the current study, we used excess copper as representative heavy metal stress and analyzed the expression profiles of *SOD SVs*.

**Figure 6:**
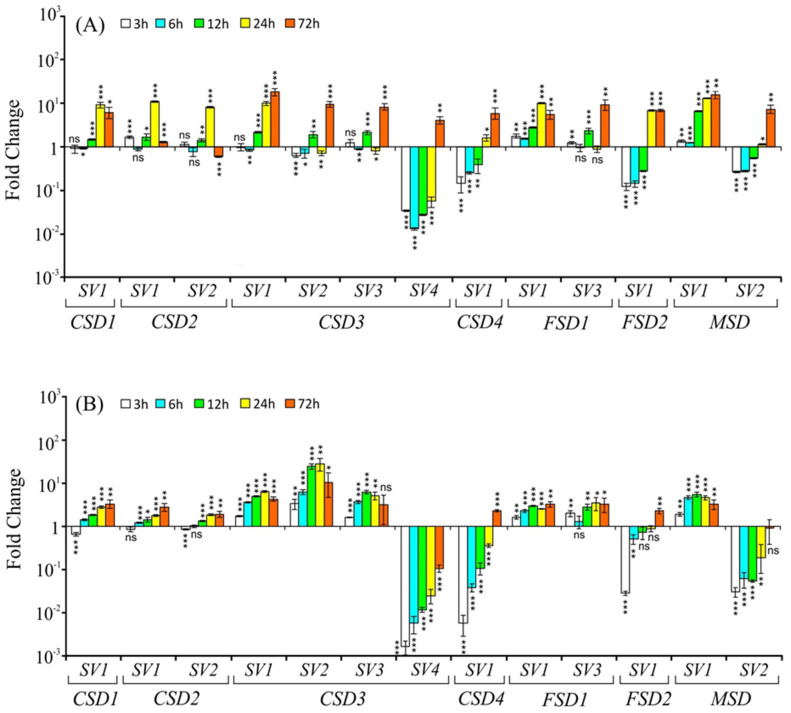
Relative transcript levels (on *Y*-axis) of *CSDs*, *FSDs*, and *MSD* splice variants (indicated by numbers on *X*-axis) in response to 100 µM Cu^2+^ stress, in shoot (**A**) and root (**B**) tissue at 3 h, 6 h, 12 h, 24 h, and 72 h after the stress treatment. For normalization and statistical analysis, see figure legend 3.

Several *SVs* showed variations in their expression profiles under high levels of Cu^+2^ (100 µM) treatment (Figure 6A,B). In shoots, the constitutive transcripts of *CSD1-SV1*, *CSD2-SV1,* and *CSD3-SV1*, were largely unaffected at 3 h and 6 h but significantly upregulated at later time points, whereas *FSD1-SV1* and *MSD-SV1* were significantly upregulated at all time points and the degree of upregulation intensified with increasing duration. By contrast, *CSD4-SV1* and *FSD2-SV1* displayed downregulation until 12 h and then upregulation at later time points (Figure 6A). The *SVs* of *SODs*, *CSD2-SV2*, *CSD3-SV2*, *CSD3-SV3,* and *FSD1-SV3* levels were either unaffected or mildly regulated during initial time points but showed significant upregulation at later time points. By contrast, *CSD3-SV4* and *MSD-SV2* levels were significantly downregulated until 24 h and then upregulated at 72 h. In general, at later time points (24 h or 72 h), both constitutive and the *SVs* were strongly induced in shoots, and several of them showed ~10-fold or even higher upregulation compared to the controls (Figure 6A).

In roots, the constitutive transcripts of five *SODs*, i.e., *CSD1-SV1*, *CSD2-SV1*, *CSD3-SV1*, *FSD1-SV1*, and *MSD-SV1*, were largely upregulated in a duration-dependent manner, mimicking the responses found in shoots (Figure 6B). In contrast, the *CSD4-SV1* levels were downregulated initially until 24 h and then upregulated at 72 h (Figure 6A). With regard to the *SVs* of *CSD2* and *CSD3* (with the exception of *CSD3-SV4*), the expression profiles largely mirrored the constitutive transcripts, although the degree of regulation was low. The transcript levels of two *SVs* (*CSD3-SV4* and *MSD-SV2*) were downregulated in contrast to the regulation of their corresponding constitutive transcripts (*CSD3-SV1* and *MSD-SV1*) under heavy metal stress.

**Figure 7:**
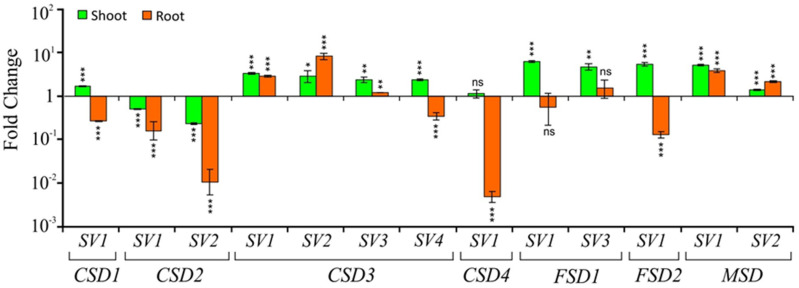
Relative transcript levels (on *Y*-axis) of *CSDs*, *FSDs*, and *MSD* splice variants (indicated by numbers on *X*-axis) in response to copper deficiency, in shoot and root tissue after 24 h of the stress treatment. For normalization and statistical analysis, see figure legend 3.

While copper in excess causes heavy metal toxicity, it is required by plants in trace amounts because it plays critical roles in photosynthetic and respiratory electron transport chains, ethylene signaling, cell wall metabolism, and several other physiological processes [30]. When considering *CSD*s, copper is even more important because it is an essential cofactor of the *CSD*s. Under copper-deprivation, the available copper is predisposed to be allocated for plastocyanin, an essential protein in the photosynthetic electron transport chain rather than to *CSD*s, thus decreasing the expression of *CSDs* at the transcriptional level [31]. In addition, *CSD*s suppression is also attained at the post-transcriptional level via miR398-mediated regulation under copper-deprivation [31,32]. By contrast, other SODs, especially FSDs, are upregulated under copper deprivation to maintain scavenging of superoxide radicals [31]. To assess the effect of copper-deprivation on the *SVs* of *SODs*, their expression profiles were analyzed after 24 h of treatment in both shoot and root tissues (Figure 7).

In shoots, interestingly, out of the four *CSD*s, levels of three *CSD*s (*CSD1*, *CSD4,* and both *SVs* of *CSD2*) were downregulated while *CSD3* and its *SVs* (*CSD3-SV1*, *CSD3-SV2*, *CSD3-SV3,* and *CSD3-SV4*) were upregulated under copper-deprivation. On the other hand, both the constitutive forms as well as their *SVs* of *FSDs* and *MSDs* were upregulated in shoots (Figure 7). In roots, *CSD1-SV1*, *CSD2 -SV1,* and *-SV2,* as well as *CSD3-SV4*, *CSD4-SV1*, and *FSD2-SV1,* levels were downregulated. Of these, maximum downregulation was observed for *CSD4-SV1* followed by both variants of *CSD2* (*SV1* and *SV2*) (Figure 7). In contrast, the constitutive form of *CSD3* (*CSD3-SV1*) and two of its *SVs* (*CSD3-SV2* and *CSD3-SV3*) as well as both the *MSD-SV1* and *MSD-SV2* levels were upregulated in roots under copper-deprivation (Figure 7). Overall, as expected, majority of the *CSDs*, *FSDs,* and *MSDs* and their *SVs* showed downregulation, whereas *CSD3* and its *SVs* displayed upregulation in both roots and shoots, although there were mild differences between the tissues.

### 2.4. The Targeting Potential of miR398 on SVs of CSDs

**Figure 8:**
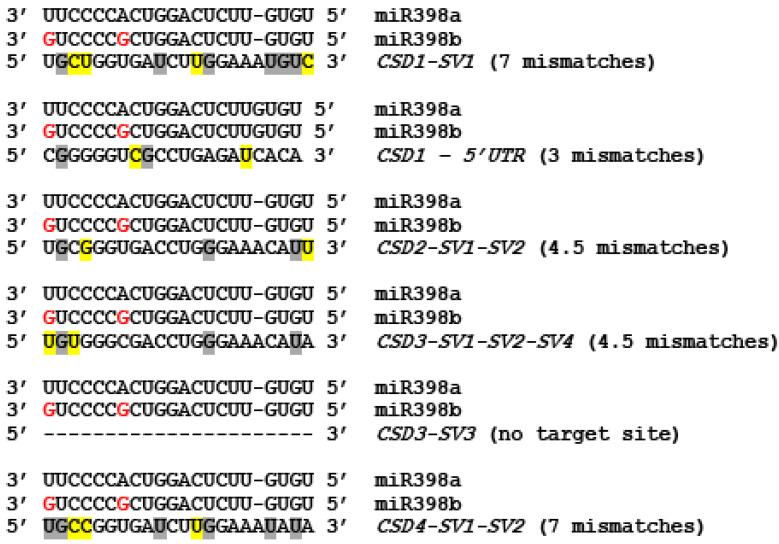
Sequence alignment of miR398 and miR398 target sites with the four rice *CuZn-SODs*. Top panel shows the miR398a and miR398b sequence and the variations (indicated in red font color). The bottom panel shows the mismatches (shaded in yellow color) and G:U wobble (shaded in grey color) in the corresponding target region of the target transcripts. Some G:U pairs are not grey shaded because those ‘G’ can be perfectly paired with ‘A’ present in one of the miR398s.

The *CSD*s are targeted by miR398 in plants including rice [27,33]. Analysis of miR398 target sites on *CSD*s, i.e., *CSD1*, *CSD2*, *CSD3*, and *CSD4*, revealed substantial differences with regard to the targeting potential based on the number of mismatches between miR398 and the *CSD*s (Figure 8). For instance, *CSD1* (at 5′ UTR), *CSD2,* and *CSD3* had 3 or 3.5 mismatches, while *CSD4* had 6 mismatches when aligned with the miR398. Among the *SVs* of *CSD3*, *CSD3-SV3* did not contain the target site for miR398, while the constitutive form of *CSD3* (*CSD3-SV1*) and other *SVs* (*CSD3-SV2* and *CSD3-SV4*) possessed miR398 target sites with 3.5 mismatches (Figure 8).

### 2.5. Characteristics of Predicted Proteins Encoded by Splice Variant Transcripts

The *SVs* of *SOD* genes (*CSD2*, *CSD3*, *FSD1*, and *MSD*) showed heterogeneity in the number and length of the exons (Figure 1), resulting in variation in length, sequence, and associated characteristics of the predicted proteins (Table 1). The predicted protein of CSD2-SV2 showed minor variation in molecular weight (MW), but the same isoelectric point (pI) values compared to the protein encoded by CSD2-SV1 (constitutive form). Due to variation in large exonic regions, the predicted proteins of CSD3-SV2, -SV3, and -SV4 differed considerably in estimated MW and pI values than the CSD3-SV1 (constitutive form) encoded protein. Similarly, predicted proteins of FSD1-SV3 and MSD-SV2 differed substantially in MW and pI values compared to the protein encoded by the corresponding *SV1′*s protein isoforms (Table 1).

**Figure 9:**
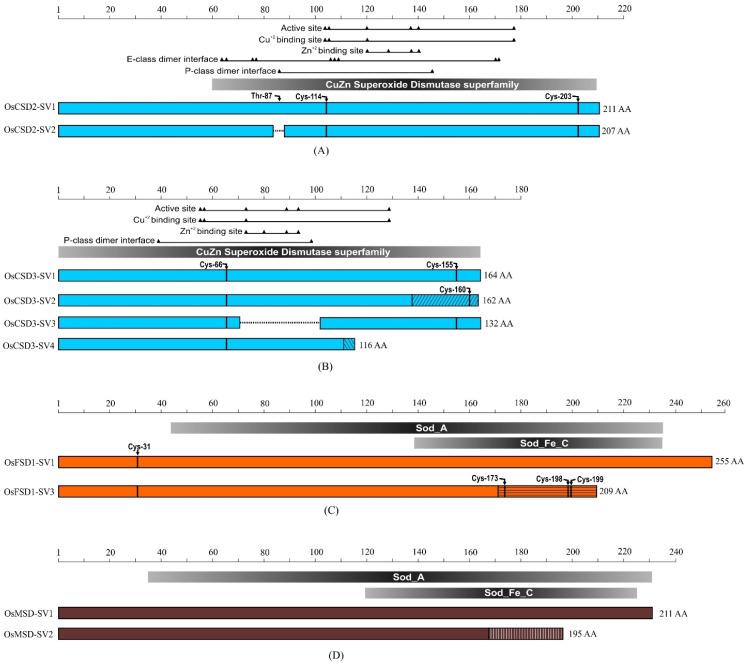
Schematic overview of the comparison of important features of putative proteins encoded by splice variants of rice *SOD*s exhibiting differences in exon combinations based on the analysis at Conserved Domain Database (https://www.ncbi.nlm.nih.gov/Structure/cdd/wrpsb.cgi) at NCBI. (**A**) Comparison of features of predicted proteins encoded by two *OsCSD2* splice variants. (**B**) Comparison of important features of predicted proteins encoded by four *OsCSD3* splice variants. (**C**) Comparison of features of predicted proteins encoded by two *OsFSD1* splice variants. (**D**) Comparison of features of predicted proteins encoded by two *OsMSD* splice variants. The three types of SODs are represented by different colors, blue (CuZn-SODs), orange (Fe-SODs), and brown (Mn-SOD). The slanting/vertical/horizontal lines indicate amino acid sequence encoded by alternative exons. The missing region in protein variants is indicated by a black dashed line. The length of the predicted proteins, domains, and positions of residues involved in active sites, metal cofactor binding, subunit interaction, and disulfide bonds are indicated.

To assess potential structural and functional differences between the constitutive proteins and their SV-encoded proteins, protein domains of the constitutive proteins (SV1s) of the CSD2, CSD3, FSD1, and MSDs and their corresponding SVs were compared using the Conserved Domain Database (CDD) at the NCBI (https://www.ncbi.nlm.nih.gov/cdd/) (Figure 9). The CSD2-SV2 encoded protein was four amino acids shorter than the CSD2-SV1 (constitutive) encoded protein (Figure 1). This small change did not lead to the loss of any important amino acid residue for metal cofactor binding or the active site, except for a single amino acid (Thr-87) involved in subunit interaction (Figure 9A). Among the three alternative transcripts of *OsCSD3*, the *SV2* encoded protein contained a C-terminal coding region encoded by an alternative exon encoding 25 amino acids. It also lacked Cys-155, which forms an intra-subunit disulfide bond with Cys-66 but contained another Cysteine at 160th position (Figure 9B).

The *CSD3-SV3* encoded protein was missing the exon-3, which contained the residues involved in binding Zn^+2^ (His-72, His-80, His-89, and Asp-92), and Val-97 important at the dimer interface (Figure 9B). Similarly, the *CSD3-SV4* encoded protein was truncated at the C-terminus and also contained few different amino acids at the C-terminal region due to an intron retention event but lacked His-129 residue (which is important for active site formation and Cu binding) as well as Cys-155 (involved in disulfide bond formation) (Figure 9B). Compared to *FSD1-SV1*, the *SV3* encoded predicted protein was found to lacking a considerable region towards the C-terminus, which was partially replaced by another sequence due to an intron retention event. This resulted in a loss of a considerable part of the SOD_A domain (or SOD_Fe_C domain) and the presence of multiple cysteine residues (Cys-173, Cys-198, Cys-199) in the replaced sequence (Figure 9C). The *MSD-SV2* encoded predicted protein also lacked a considerable region at the C-terminus, which resulted in a partial loss of the SOD_A domain (or SOD_Fe_C domain) (Figure 9D). The predicted alternative proteins encoded by the splice variants revealed differences in specific characteristics of the protein sequence.

## 3. Discussion

Alternative splicing is a complex process, regulated by several factors at different steps, including those involved in recognition of correct splice junctions to channelize the transcript towards constitutive or alternative fates in different tissue or at developmental stages or under stress conditions, including stress-memory in plants [6,9,34,35]. The RGAP database predicts that the rice *SOD*s undergo AS and produce seven *SV*s by utilizing intron retention (*CSD3-SV4* and *FSD1-SV3*), exon skipping (*CSD3-SV3* and *MSD-SV2*), alternative splice junctions (*CSD3-SV2*), and alternative 5′-splice sites (*CSD4-SV2* and *CSD2-SV2*). Our transcript expression profiles under diverse stress conditions have demonstrated that all *SVs* of *SOD*s in rice were affected by abiotic stresses. Compared to their constitutive transcripts, the *SVs* of certain *SODs* showed opposite profiles in different tissues (shoots or roots) in a stress-specific manner, suggesting a potential role for the SVs under stress conditions.

The present study shows that the predicted proteins encoded by *SOD SVs* with the variable exonic organization have different features (missing Zn binding residues, surface interacting residues, and extra/missing cysteine residues, Figure 9) that are likely to affect the structure, function, and properties of the isoforms. Thus far, an SV of Fe-SOD is the only cloned SV from the rice SOD family, which was shown to be enzymatically active in vitro [21]. Similarly, recently identified *SV* of *FSD3* (*FSD3*s) in Arabidopsis retained two introns (6 and 7) but included a stop codon at an earlier position than the constitutive *FSD3.* However, the FSD3s was functional, suggesting that the C-terminal region is not required for its activity [24]. The FSD3 is a chloroplast localized protein in Arabidopsis. Within the chloroplast, the localization pattern of SV FSD3s (thylakoid membrane) differed from the FSD3 (chloroplast nucleoid) [24].

It is known that the catalytic activity of CuZn-SODs is driven by the redox-active Cu, while Zn is important for stabilizing the protein but can also enhance the catalysis. Interestingly, OsCSD3-SV3, one of the SVs of OsCSD3, lacks a Zn-binding site (Figure 9B). Previously, the Cu-only containing SODs have been reported from bacteria (Mycobacteria) and fungi, and these SODs can protect fungi from the fungal pathogen-induced oxidative stress [36]. Further studies are needed to determine whether Zn-lacking OsCSD3-SV3 is enzymatically active in rice. The rice Fe-SOD-SV3 (FSD1-SV3) lacking 46 amino acids at the C-terminus is yet another worth mentioning SV (Figure 9C). Recently it was shown that the Arabidopsis FSD3s was active despite lacking the C-terminal region [24]. On the other hand, isoforms with variations in their UTRs may affect the stability of the transcripts. It was shown that the differences generated by AS in the UTRs may alter mRNA transport, stability, or translational regulation [2,4]. Taken together, these observations suggest that many of the SVs of SODs in rice could be functional; therefore, they could be important under abiotic stress conditions.

The regulation of gene expression in response to stress conditions in plants is controlled largely at the transcriptional level, but post-transcriptional regulation also plays an important role. At the post-transcriptional level, the *SODs*, particularly *CSDs,* are likely to be regulated by miR398, which can guide transcript cleavage and/or translational inhibition, and this regulation appears to be important for stress responses [32]. On the other hand, under copper-deprivation, the miR398-mediated downregulation of CSDs enables the plants to channel low amounts of available copper to photosynthetically important proteins [37]. The alignment of four transcripts of rice *CSDs* with miR398 revealed differences in number of mismatches (Figure 8), suggesting that miR398-mediated regulation of different CSDs is likely to differ. The *CSD1* and *CSD2* are known to be regulated by miR398, but our study predicts that the *CSD3* transcripts are also likely to be targeted by miR398 in rice. Intriguingly, *CSD4* that possess the maximum number of mismatches with miR398 showed strong downregulation under copper-deprivation compared to the *CSD1* and *CSD2* that have a smaller number of mismatches. However, such differences could also be attributed to the transcriptional regulation. Additional studies are needed to determine the role of miR398 in regulating these different *SVs* of *CSDs* in rice.

Overall, the expression profiles of the majority of the *SVs* both in shoots and roots mimicked their corresponding constitutive *SOD* transcripts under stress conditions. Nevertheless, inverse profiles were observed for specific *SVs* compared to their constitutive forms. For instance, in roots, *CSD3-SV4* consistently displayed opposite profiles under salt stress, osmotic stress, cold stress, and heavy metal stress, as well as under copper-deprivation compared to its constitutive form (*CSD3-SV1*) (Figure 3, Figure 4, Figure 5, Figure 6 and Figure 7). Similarly, also in shoots, the *CSD3-SV4* profiles differed compared to its constitutive form (*CSD3-SV1*), although the differences were not as distinct as that were observed in roots. Notably, *CSD3-SV4* in shoots showed similar regulation like that of the constitutive transcript (*CSD3-SV1*) under cold stress suggesting that the regulation of *SV* is stress-specific and tissue-specific. *MSD-SV2* is another *SV* that showed opposite profiles at specific time points of the treatment and under certain stress conditions compared to its constitutive form, *MSD-SV1*. In roots, *MSD-SV2* profiles were largely downregulated under salt, cold, and heavy metal stresses, while *MSD-SV1* levels were often upregulated. In shoots, the differences were somewhat less consistent across the stress conditions and time points but displayed opposite profiles. Taken together, these observations suggest that the AS of *SOD*s is an active process, and specific SVs are likely to be important in a tissue-specific and stress-specific manner.

In addition to post-transcriptional regulation, SODs are known to undergo post-translational modifications, which could affect the function of the *SOD*s. For instance, phosphorylation at serine/threonine residues of SOD1 (Cu/Zn SOD) in humans can affect degradation or subcellular localization [38]. In both yeast and humans, Dun1 kinase-mediated phosphorylation of S60 and S99 in response to oxidative stress caused by H_2_O_2_ treatment can lead to the translocation of SOD to the nucleus for transcriptionally activating oxidative stress resistance and DNA repair genes [39]. On the other hand, acylation on lysine residues (1-Carbon acetylation of Lys70; 2-Carbon succinylation of Lys122) could inactivate the SOD activity; lysine glycosylation can cause a loss of SOD activity; sumoylation or ubiquitination at lysine, S-acylation (lipid attachment via a thioester bond) at cysteine side chains, nitration at tryptophan residues have also been identified for human SOD1 protein; oxidation of Cys111 to sulfonic acid leads to misfolding and aggregation; glutathionylation of Cys111 destabilizes SOD and promotes monomer formation [38]. By contrast, cysteinylation at Cys111 protects SOD from oxidation and aggregation [38]. Compared to animal systems, the PTM of plant SODs are relatively less understood. In Arabidopsis, NO (nitric oxide)-dependent post-translational modifications (nitration–ONOO^-^) of tyrosine residues was shown to inhibit peroxisomal CSD3 (30% inhibition), chloroplastic FSD3 (30% inhibition), and mitochondrial MSD1 (90% inhibition) activity [40], suggesting that the SODs in plants also undergo PTMs which could affect the enzyme activity since PTMs are dependent on the presence or absence of specific amino acid residues, and AS could remove specific exons containing such residues, which, in turn, could affect the function of SOD isoform. Future studies should evaluate the importance of PTMs in AS-generated isoforms lacking these modifications and their roles in structure/function of specific SOD isoforms.

In addition to known regulatory mechanisms, such as the transcriptional and miRNA-mediated post-transcriptional regulation, the present study revealed that the SODs in rice undergo AS, adding further complexity to their regulation both under normal and stress conditions. Nevertheless, the present study cannot rule out the possibility that some of these SVs of SODs could be non-functional. Further studies are required to determine the activity of SVs of SODs and their importance in tissue-specific and stress-specific processes.

## 4. Materials and Methods

### 4.1. Plant Material and Growth Conditions

Seeds of *Oryza sativa* (cv. Nipponbare) were surface sterilized with 20% bleach and germinated on sterilized wet filter paper at 37 °C. The germinated seedlings were transferred to 96-well PCR microplates, which were precut at the bottom, and the plates were allowed to float on Yoshida liquid media [41]. The plates containing seedlings were kept in a growth chamber that was maintained at 29 °C/22 °C (day/night temperatures), with 12 h of photoperiod. The liquid media was regularly replaced on alternate days. Fifteen-day-old seedlings were subjected to salinity (150 mM NaCl), osmotic stress (15% PEG-6000), cold stress (4°C), and heavy metal (Cu^+2^) stress, including copper deprivation. Shoot, and root samples from the control and stress-treated seedlings were collected separately at five different time points (3 h, 6 h, 12 h, 24 h, and 72 h) and were snap-frozen in liquid nitrogen and stored at −80 °C until further use. The seedlings exposed to copper-deprivation were harvested at the 24 h time-point only.

### 4.2. Identification of Splice Variants

The nucleotide and amino acid sequences of the SVs of rice CuZn-SOD, Fe-SOD, and Mn-SODs were retrieved from the Rice Genome Annotation Project database, Release 7 (http://rice.plantbiology.msu.edu/; Kawahara, et al. [42]). A theoretical molecular weight (MW) and isoelectric point (pI) of the sequences were estimated by ‘Compute pI/Mw tool’ tool at the ExPASy website (http://web.expasy.org/compute_pi/). Alternative transcript and protein sequences were aligned by ClustalX software [43] using default gap opening and gap extension penalties and further edited by BioEdit Software [44]. Putative protein domains were identified using the conserved domain search (CD-Search) function at Conserved Domain Database web server (https://www.ncbi.nlm.nih.gov/Structure/cdd/wrpsb.cgi; Marchler-Bauer et al. [45]) at the National Center for Biotechnology Information website (NCBI, https://www.ncbi.nlm.nih.gov).

For each gene, the constitutive transcript was considered as the first splice variant and designated as SV1, and their SVs were termed as SV2, SV3, and SV4 (Figure 1). Thus, the canonical transcripts of rice *CSDs* were denoted as *OsCSD1-SV1*, *OsCSD2-SV1*, *OsCSD3-SV1,* and *OsCSD4-SV1*. Of these, *CSD1* was not found to undergo AS, while the remaining three *CSDs* generated SVs, and their SVs were symbolized as *OsCSD2-SV2*, *OsCSD3-SV2*, *OsCSD3-SV3*, *OsCSD3-SV4,* and *OsCSD4-SV2*. Similarly, the canonical transcripts of *FSDs* were designated as *OsFSD1-SV1* and *OsFSD2-SV1,* whereas their SVs were shown as *OsFSD1-SV3* (*OsFSD2* did not undergo AS). The *OsMSD-SV1* was the canonical transcript for *MSD,* and its SV was denoted as *OsMSD-SV2*.

### 4.3. RNA Isolation, Reverse Transcription, and RT-qPCR Analysis

Total RNA was isolated from the rice tissues using TRIzol reagent (Invitrogen, USA), and two micrograms was initially treated with DNase I and then reverse-transcribed using Superscript-II reverse transcriptase (Invitrogen, USA) as per the manufacturer’s recommendations. First-strand complementary DNA (cDNA) was used for quantitative RT-PCR (RT-qPCR) analysis. RT-qPCR analysis was carried out using primers designed for specific amplification of the *SOD* SVs (Table S1). In general, the primers were designed for the regions (exons or UTRs) that were either present or absent among the splice variants. Furthermore, at least one primer in a pair was designed in the exon-exon or exon-UTR junction wherever it was feasible (*CSD2-SV1* and -*SV2*, *CSD3-SV4*, *CSD4-SV1* and -*SV2*, *FSD1-SV3*, *MSD-SV1* and -*SV2*, Figure 1). RT-qPCR analysis was carried out using Maxima^TM^ SYBR Green qPCR Master Mix (ThermoFisher Scientific, USA) on a Real-Time PCR System (model 7500 Applied Biosystems, Foster City, CA, USA) using 200 ng cDNA and 7.5 picomoles of each of the SV-specific primers. In the case of low transcript levels, 500 ng cDNA was used for the analysis. The thermal cycling conditions for the two-step PCR program used during RT-qPCR analysis were as follows: initial denaturation at 94 °C (10 min), 45 cycles of 94 °C (10 s), 60 °C (50 °C for OsCSD4-SV4, 20 s), and 65 °C (35 s). The PCR cycling was followed by melting curve analysis to assess the specificity of the amplification. The amplicons were also analyzed on agarose gel. RT-qPCR analysis was carried out using two independent biological replicates, where 15–20 rice seedlings were pooled for RNA extraction followed by cDNA synthesis in each sample. All PCRs were run three times and with appropriate controls, including a non-template control (NTC) for each set of the analyses. The transcript levels were normalized against OsActin in control and stress-treated samples, and the fold change (relative expression) was estimated using the 2^−∆∆Ct^ method [28,46]. Rice actin-2 (LOC_Os10g36650) was used as a reference gene for the RT-qPCR analyses. Statistical analysis of the RT-qPCR data was carried out using GraphPad Prism (version 7) software (GraphPad Software Inc., San Diego, CA, USA). Normalized transcript levels between control and treated samples were compared by applying *t*-tests, and level of significance is indicated by the following symbols: ns (no significant difference), * (*p* < 0.05), ** (*p* < 0.01), *** (*p* < 0.001).

## Figures and Tables

**Table 1:** Characteristics of coding region and predicted amino acid sequence of rice CuZn, Fe and Mn superoxide dismutases (SODs) and their splice variants.

^a^ Genomic Locus and the SOD Designation	Splice Variant	Size of Coding Region (bp)	Number of Amino Acids	^b^ Subunit Molecular Weight (Da)	^b^ Iso-Electric Point (pI)	^c^ GenBank Rice EST Database Results
LOC_Os03g22810 (OsCSD1)	SV1	813	270	27,868.14	6.25	>30 ESTs (Cov: >98%, Id: >99%)
LOC_Os08g44770 (OsCSD2)	SV1	636	211	21,300.99	5.79	2 ESTs (CI026601, CT842196)
	SV2	624	207	20,944.61	5.79	1 EST (CI764803)
LOC_Os03g11960 (OsCSD3)	SV1	495	164	16,517.36	6.82	8 ESTs (Cov: >98%, Id: >98%)
	SV2 ^#^	489	162	16,821.86	6.01	1 EST (CI239237)
	SV3 ^#^	399	132	13,102.74	7.91	2 ESTs (CI026601, CT842196)
	SV4 ^#^	351	116	11,782.04	6.41	2 ESTs (CX728298, CB679877)
LOC_Os07g46990 (OsCSD4)	SV1	459	152	15,080.68	5.92	16 ESTs (Cov: 100%, Id: >99%)
	SV2 ^#^	459	152	15,080.68	5.92	>10 ESTs (Cov: >80%, Id: >97%)
LOC_Os06g05110 (OsFSD1)	SV1	768	255	29,476.58	8.84	2 ESTs (Cov: >93%, Id: >94%)
	SV3^#^	630	209	24,182.73	9.01	5 ESTs (Cov: 100%, Id: 100%) AY770495 [21]
LOC_Os06g02500 (OsFSD2)	SV1	1176	391	43,420.42	5.45	2 ESTs (Cov: >70%, Id: >96%)
LOC_Os05g25850 (OsMSD)	SV1	696	231	24,997.48	6.50	5 ESTs (Cov: 100%, Id: >99%)
	SV2 ^#^	588	195	20,827.06	6.49	1 EST (CI243477)

^‘a’^ Genomic locus is as per the information at Rice Genome Annotation Project website (http://rice.plantbiology.msu.edu/). ^‘b’^ Isoelectric point (pI) and molecular weight (MW) of protein sequences were estimated using the ‘Compute pI/Mw software tool’ at ExPASy (http://www.expasy.ch/cgi-bin/pi_tool). ‘^#^’ Splice variant sequence that showed variation in the untranslated regions (UTRs). ^‘c’^ The splice variant sequences were searched against GenBank (EST) database (organism: ‘rice’ specific query). In general, splice variant specific regions (UTR or exons with a flanking region) were used as the query in the Blastn program. Hits with high coverage (Cov) and identity (Id) were recorded and listed. Low coverage observed sometimes resulted from the partial EST sequences in the database. Blastn search was also carried out in GenBank ‘nr’ database, which resulted in more hits than the ‘EST’ database search.

## Data Availability

Not applicable.

## Supplementary Materials

The following are available online at https://www.mdpi.com/article/10.3390/ijms22083997/s1, Figure S1: Analysis of PCR amplified SOD splice variant (SV) fragments using variant-specific oligonucleotide primers on a 2.5% agarose gel, Table S1: The oligonucleotide sequence of the primers used for RT-qPCR analysis.
