# Supplementary material for: Splice Variants of Superoxide Dismutases in Rice and Their Expression Profiles under Abiotic Stresses
Source: Int J Mol Sci. 2021 Apr 13;22(8):3997. doi: 10.3390/ijms22083997 (PMC8068833; doi:10.3390/ijms22083997)
Supplement: Supplementary file 1 [file ijms-22-03997-s001.pdf]

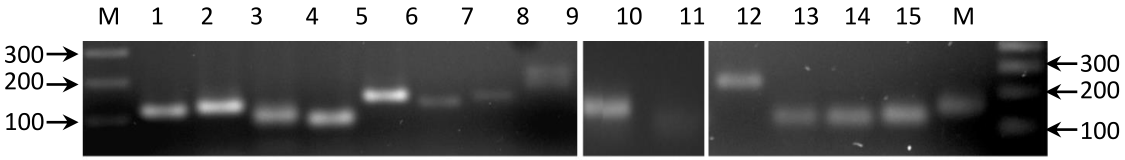

Figure S1: Analysis of PCR amplified SOD splice variant (SV) fragments using variant specific oligonucleotide primers on a 2.5% agarose gel. Lane 1) Actin, lane 2) *OsCSD1-SV1*, lane 3) *OsCSD2-SV1*, lane 4) *OsCSD2-SV2*, lane 5) *OsCSD3-SV1*, lane 6) *OsCSD3-SV2*, lane 7) *OsCSD3-SV3*, lane 8) *OsCSD3-SV4*, lane 9) *OsCSD4-SV1*, lane 10) *OsCSD4-SV2*, lane 11) *OsFSD1-SV1*, lane 12) *OsFSD1-SV3*, lane 13) *OsFSD2-SV1*, lane 14) *OsMSD-SV1*, and lane 15) *OsMSD-SV2*. Lane M indicates 100 bp ladder.

Table S1: The oligonucleotide sequence of the primers used for the RT-qPCR analysis.

Gene & transcript designation[#]	Gene locus[*]	Forward oligonucleotide primer	Reverse oligonucleotide primer	Amplicon size (bp)
OsActin	LOC_Os10g36650	5'- CTAGTGGACGTACTACTGGTATTG -3'	5'- GATCCCTACCAGCAAGATCAAGAC -3'	123
OsCSD1-SV1	LOC_Os03g22810	5'- GGAGAAGATGGTGTTGCTAATATCCA -3'	5'- TCGTGCCCACCCTTTCCAAGAT -3'	128
OsCSD2-SV1	LOC_Os08g44770	5'- AAGAAGGCCGTCGCCGTGCTCAA -3'	5'- CACGGACATTCACTGTTGTAGGACCT -3'	97
OsCSD2-SV2		5'- AAGAAGGCCGTCGCCGTGCTCAA -3'	5'- GTCCCGTCACACGGACATTCACTT -3'	94
OsCSD3-SV1	LOC_Os03g11960	5'- GTAGCCAACAAAGATGGTGTTGCA -3'	5'- TGTTTTACTGAGTTCATGACCACC-3'	147
OsCSD3-SV2		5'- GATGACCTAGGAAGGGCTATGGT -3'	5'- AGTTCATGACCACCTGTCTTATGA-3'	129
OsCSD3-SV3		5'- GCAACTCTACCGGTGTTGCAG-3'	5'- TGTTTTACTGAGTTCATGACCACC-3'	143
OsCSD3-SV4		5'- ACGGCTGCAACTCTACCGGGCC-3'	5'- ATATTTTACATATTTCAAGACAATGGA -3'	175
OsCSD4-SV1	LOC_Os07g46990	5'- CCTTCTGGAGTCTTCCTCATCAGAA -3'	5'- GCAACAGCCTTCACCATTGTCTATGTGTTCTC -3'	126
OsCSD4-SV2		5'- CCTTCTGGAGTCTTCCTCATCAGAA -3'	5'- AGCAACAGCCTTCACCATCTCA -3'	115
OsFSD1-SV1	LOC_Os06g05110	5'- GACTTGTGGGAGCATGCTTACTAC -3'	5'- GAAATTCCTCGGCGTCGCTCATGA -3'	235
OsFSD1-SV3		5'- TCTAGACTTGTGGGAGGTAAGGA -3'	5'- CATTCGCCTGTCATCCTTGTAATC -3'	125
OsFSD2-SV1	LOC_Os06g02500	5'- GGTAGACTGGTCATCTCAAAGTCT -3'	5'- GTCAGATCTCCGATCCTCATAATC -3'	126
OsMSD-SV1	LOC_Os05g25850	5'- CAGTGGAAACAACTGCTAACCAGG -3'	5'- GTTGCTCAGGTAGTCTGGCCTGAC -3'	134
OsMSD-SV2		5'- TGGTGGTGATCCACCACATGCAA -3'	5'- ACCATGAGCGTTGCCTCTTTATCC -3'	156

'#' Gene & transcript designation: CSD: CuZn Superoxide dismutase, FSD: Fe Superoxide dismutase, MSD: Mn Superoxide dismutase; SV: Splice variant; Numerals 1, 2, 3 and 4 along with 'SV' refers to alternative transcripts where the SV1 is always the constitutive variant and subsequent numbers are the alternate variants.

'*' Gene locus is the chromosomal locus designation as per the Rice Genome Annotation Project database Release 7 (http://rice.plantbiology.msu.edu/)
